# Citrate anticoagulation for continuous venovenous haemodiafiltration: the impact of a novel protocol on patients receiving therapy in one regional hospital

**DOI:** 10.1186/cc14382

**Published:** 2015-03-16

**Authors:** J Highgate, G Escott, A Lowe, F Stedman, N McNeillis

**Affiliations:** 1 Worthing Hospital, Worthing, UK; 2 Conquest Hospital, Hastings, UK

## Introduction

Citrate has been used to anticoagulate extracorporeal haemofiltration circuits since the 1960s, and has been used as the first-line anticoagulant for continuous venovenous haemodiafiltration (CVVHDF) at Conquest Hospital since 2009. Benefits of citrate demonstrated in clinical trials include increased filter life and increased bicarbonate formation from metabolism of the citrate complex; citrate also lacks the increased bleeding risk associated with unfractionated heparin use. One of the main issues with new renal replacement therapies is the development of ideal dialysate fluids. During the initial period of citrate use at Conquest, hyponatraemia was identified as an issue, with off-license supplementation of dialysate fluid with sodium bicarbonate often necessary to prevent this. New protocols were therefore developed, designed to maximise the filtration dose and maintain normal electrolyte balance.

## Methods

A comparison of patients receiving CVVHDF on the 11-bed critical care unit at Conquest Hospital, Hastings was undertaken, before and after the implementation of new CVVHDF protocols. All patients receiving CVVHDF were identified from the electronic patient record system between March 2012 to 2013 and September 2013 to 2014. Patient demographics, the duration of CVVHDF and sodium bicarbonate supplementation were analysed between the groups to assess the impact of the new protocols.

## Results

Sixty-four patients received CVVHDF in 2012 to 2013, 61 receiving citrate and three receiving unfractionated heparin due to fulminant liver failure. Forty-seven patients received CVVHDF in 2013 to 2014, two receiving no anticoagulation due to severe coagulopathy and one receiving unfractionated heparin. The two patient cohorts assessed were similar in age (median 65.5 for March 2012 to 2013 cohort vs. 66 for September 2013 to 2014 cohort), gender mix (64% male vs. 57% male) and severity of illness as assessed by APACHE II score (23 vs. 24). Mean duration of CVVHDF was also similar (71.5 hours vs. 75 hours). A total 30/64 of 2012 to 2013 patients did not require a filter change prior to completion of RRT, compared with 23/47 of 2013 to 2014 patients. Sodium bicarbonate was added to the dialysate fluid in 29/64 2012 to 2013 patients, compared with just 2/47 2013 to 2014 patients.

## Conclusion

Changing protocols resulted in a significant reduction in off-license addition of sodium bicarbonate to dialysate bags without impacting on filter life, thus reducing nursing workload and removing a potential source of adverse events in this high-risk group of patients.

